# Role of LDL-C level alteration in increased mortality risks in spontaneous intracerebral hemorrhage patients: Systematic review and meta-analysis

**DOI:** 10.3389/fneur.2023.1114176

**Published:** 2023-02-28

**Authors:** Jing Li, Gang Li, Yajun Zhu, Xingwei Lei, Guihu Chen, Jiachun Zhang, Xiaochuan Sun

**Affiliations:** Department of Neurosurgery, The First Affiliated Hospital of Chongqing Medical University, Chongqing, China

**Keywords:** low-density lipoprotein cholesterol, intracerebral hemorrhage, mortality, meta-analysis, systematic review

## Abstract

**Background:**

Current studies indicate a contradictory relationship between decreased mortality risks of spontaneous intracerebral hemorrhage (sICH) and elevated low-density lipoprotein cholesterol (LDL-C) levels. Thus, this meta-analysis was designed to examine the involvement of high LDL-C levels in a lower mortality risk of sICH patients.

**Methods:**

PubMed, Cochrane, and Embase databases were searched up to the date of August 3rd, 2022. Pooled odds ratio (OR) with a 95% confidence interval (CI) was estimated for the higher vs. lower serum LDL-C level groups. Subgroup and sensitivity analyses were also carried out. Egger's test was applied to detect any potential publication bias.

**Results:**

Of 629 citations reviewed, 8 eligible cohort studies involving 83,013 patients were enrolled in this meta-analysis. Compared with lower serum LDL-C levels containing patients, higher serum LDL-C patients exhibited significantly decreased risks of 3-month mortality (OR: 0.51; 95%CI: 0.33–0.78; I^2^ = 47.8%); however, the LDL-C level change wasn't significantly associated with in-hospital mortality risks (OR: 0.92; 95%CI: 0.63–1.33; I^2^ = 91.4%) among sICH subjects. All studies included were classified as high-quality investigations.

**Conclusions:**

This meta-analysis suggests a higher LDL-C level may decrease the mortality risk in sICH patients. LDL-C level increase is inversely associated with the 3-month mortality risks in these patients but not significantly correlated with the in-hospital mortality risks. Further well-designed prospective studies with extended follow-up periods are needed to confirm these findings and explore underlying cross-talks.

**Systematic review registration:**

https://www.crd.york.ac.uk/prospero/display_record.php?ID=CRD42022318318, identifier: PROSPERO 2022 CRD42022318318.

## 1. Introduction

Spontaneous intracerebral hemorrhage (sICH) remains the most devastating form of stroke with high morbidity and mortality rates, accounting for over 10% of all stroke cases, and is estimated to affect nearly 2 million people worldwide each year (1–3). Despite many large-scale clinical trials and deeper insights into stroke mechanisms, proven effective therapies to ameliorate post-sICH consequences are yet to come to clinics (4, 5). sICH occurrences are multi-factorial, and prognoses are challenging in most cases. Presently, ICH management is executed primarily focusing on the risk factors manipulation, medical measures to minimize post-hemorrhage adverse consequences, and surgical interventions in certain cases (6). Therefore, efforts are continuously made to re-evaluate risk factors for stratifying mortality risks of sICH patients and improving their prognosis predictions.

Low-density lipoproteins (LDLs) are the primary transporters of cholesterol across cells and tissues, contributing to atherosclerotic lesions of the blood vessel. Studies have consistently indicated that a high LDL-C level can elevate the risks of ischemic stroke and coronary heart dysfunctions, and the causal association between these two factors is well recognized (7–9). Interestingly, LDL-C seems to have opposite effects on the risk of ischemia and sICH, which may be protective against sICH (10). Some Mendelian randomization (11, 12) and meta-analyses (13–15) demonstrate that increased LDL-C levels can lower the risk of sICH. Nonetheless, several other epidemiological investigations could not consistently show the exact implication of higher LDL-C levels on sICH mortality (16–19). To obtain more comprehensive and objective insights into the outcome and prognosis of sICH subjects with higher LDL-C levels, we conducted this meta-analytical investigation, unveiling a significant association of baseline serum LDL-C level with mortality risks in sICH.

## 2. Methods

### 2.1. Search strategy

A systematic literature search was performed in the PubMed, Cochrane Library, and EMBASE databases for studies published up to August 3, 2022, by the pre-established search strategy, using the keywords “cerebral hemorrhage,” “ICH,” “intracerebral hemorrhage,” “low-density lipoprotein cholesterol,” and “LDL-C.” The searches were conducted without language restrictions and adapted for each electronic database. The specific terms used for searching in each database, along with the number of records retrieved, are detailed in Supplementary Tables S1–S3. Besides, reference lists of retrieved articles were further screened to identify any eligible studies that didn't come up on the initial search.

### 2.2. Inclusion/exclusion criteria

Eligible studies satisfied the following selection criteria: (1) either retrospective or prospective cohort studies in nature; (2) enrolled sICH patients who were diagnostically confirmed by computed tomography (CT) or magnetic resonance imaging (MRI) examinations; (3) assessed the correlation between baseline serum LDL-C levels and the mortality risks in sICH patients, and (4) provided with univariate or multivariate-adjusted effect estimates [odds ratio (OR) with corresponding 95% confidence interval (CI)] for the association between LDL-C levels and sICH risks of mortality. The studies were excluded if: (1) these were either reviews, letters to the editor, comments, or meeting abstracts; (2) they included ICH participants with primary traumatic injuries; and (3) these were either duplicate publications or multiple articles based on the same cohort studies with overlapping data. In this case, the one with the most comprehensive results, or the largest sample size, was included.

### 2.3. Data extraction and quality assessment

Two investigators (JL and GL) independently searched and identified eligible literature for extracting relevant data for the meta-analysis, per the pre-determined selection criteria and data extraction strategies concerning the PRISMA recommendations (#PROSPERO CRD42022318318) as illustrated in Supplementary Table S5 (20). The extracted features included the first (co)author name(s), year of publication, study area and design, demographics of participants (gender variation, sample sizes, and mean/range of ages), clinical characteristics (scores on the NIHSS and GCS scales, and ICH volumes), outcome measurements, adjusted ORs with 95%CIs, and adjusted parameters in the multiple factor analysis (MFA). Once completed, investigators exchanged their data audit forms, and if there was any discrepancy, a group discussion was conducted to arrive at a consensus.

The Newcastle-Ottawa Scale (NOS) rating was applied to assess the methodological qualities of eligible articles (21). The scale includes three subscales of subject selection, comparability across groups, and ascertainment of exposure (Supplementary Table S4). Nine NOS stars referred to the maximum score for each article, of which 7–9 NOS stars corresponded to high, 4–6 stars to moderate, and ≤3 stars to low quality.

### 2.4. Statistical analyses

The ORs and 95%CIs were computed to measure the effects of altered LDL-C levels on mortality risks in sICH patients. The random-effect model (REM) was executed for meta-analyses. The Cochran's *Q*-test and I^2^ index were used to determine the heterogeneity across studies (22). Results with I^2^ values of > 50% or *P*-values < 0.05 indicated substantial heterogeneity. Subgroup analyses were performed by grouping studies based on the study area (e.g., China vs. Western) and design (prospective clinical study, PCS, vs. retrospective clinical study, RCS). The sensitivity analysis was conducted by sequential dropping of individual studies to measure the contribution of the respective study to the overall risk assessment. Egger's test was employed to determine any potential publication bias of enrolled studies (23). Stata v12.0 (Stata Corp., USA) was used for all statistical analyses.

## 3. Results

### 3.1. Literature search

**Figure 1:**
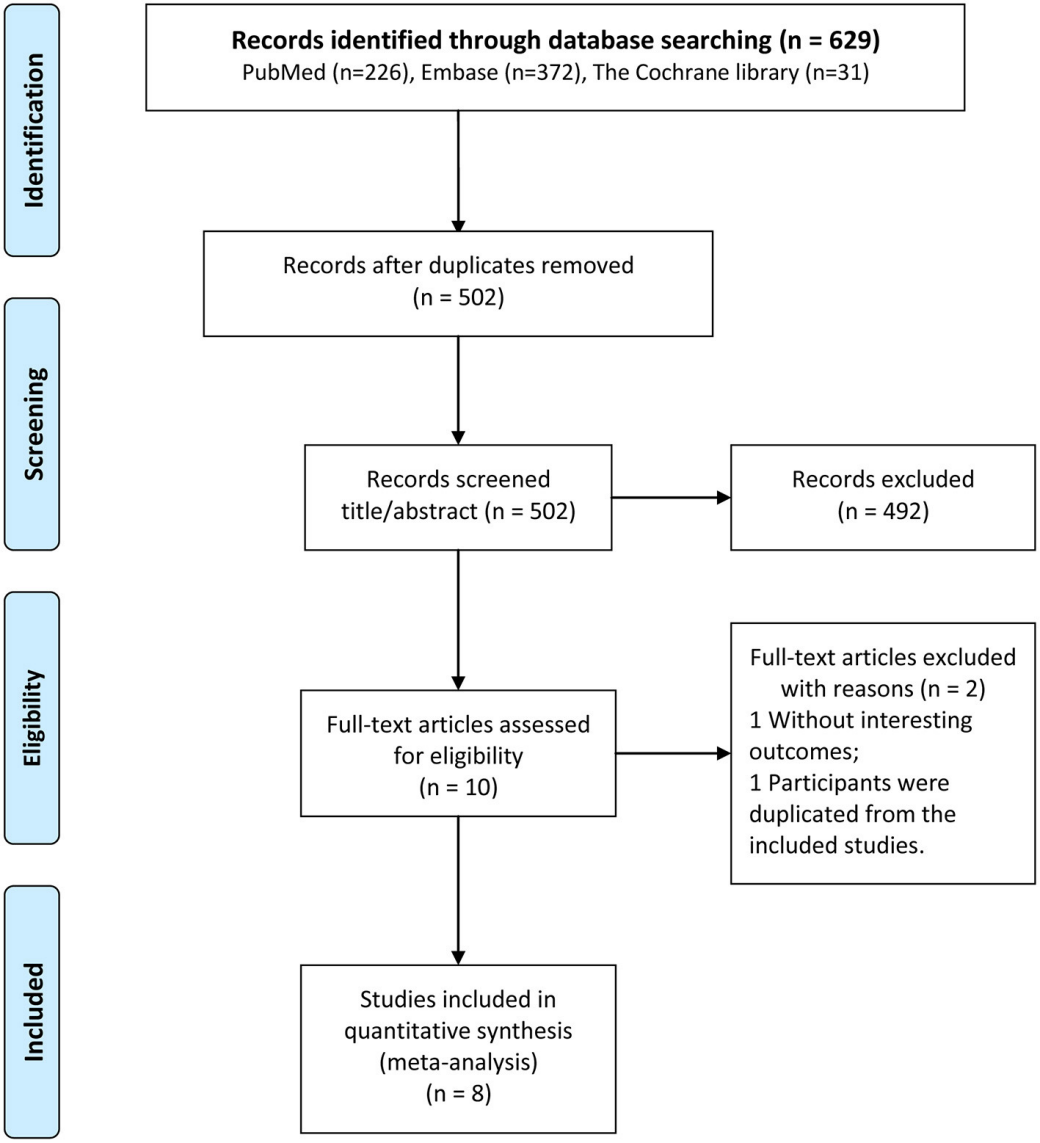
Flowchart of the selection process of included studies.

The results of the literature search and the literature screening process are shown in Figure 1. Full-text documents were retrieved for 629 articles (226 in PubMed, 372 in EMBASE, and 31 in Cochrane) from electronic databases for meta-analyses. No additional eligible study could be identified in the reference lists of included studies. After eliminating 127 duplicate articles, 502 studies were obtained in the initial screening. Of these, 492 irrelevant articles were excluded by reviewing their titles and abstracts. Then, 10 articles were subjected to full-text evaluation. Finally, 8 cohort studies (16–19, 24–27) were selected for the analysis.

### 3.2. Study characteristics

Table 1 summarizes the baseline characteristics of eligible studies. Eight selected articles published between 2009 and 2022 were analyzed. Four studies were from China (17, 25–27), and the rests were from the USA (16), Finland (18), and Spain (19, 24). There were two retrospective and six prospective cohort studies, having sample sizes ranging from 88 to 75,433 and a total of 83,013 participants. The study subjects were all acute sICH patients diagnosed by imaging methods such as CT or MRI. For each included study, the subjects' ages were in the range of 59 to 74 years. Additionally, the in-hospital mortality risk was reported in 4 studies (16–18, 26) and 5 studies (18, 19, 24, 25, 27) documented the 3-month mortality risk in sICH patients. The lower or higher LDL-C level cutoff values varied obviously across the original studies. Multivariate analyses revealed the association between LDL-C level changes and ICH mortality risks. The NIHSS and GCS scores, ICH volumes, OR (95%CI), and adjusted factors are listed in Table 1.

**Table 1:** Characteristics of eight included studies in this meta-analysis.

**Study (Area, design)**	**n, M/F**	**Age, years**	**Admission NIHSS score**	**Admission GCS score**	**ICH volume, ml**	**Exposure of LDL-C, mg/dL**	**Mortality**	**OR (95%CI)**	**Adjusted factors**
Chang et al. (16) (USA, PCS)	672, 379/293	61.6 ± 14.0	8 (2,18)	NR	NR	Per 10 unit increase	In-hospital	0.68 (0.57, 0.80)	BMI, Hypertension, Hyperlipidemia, CAD, CHF, CKD, Smoking, Admission glucose/HDL-C/Creatinine/SBP/NIHSS
Ding et al. (17) (China, PCS)	75433, 47079/28354	63.0 ± 12.8	NR	14 (8,15)	NR	>100 vs. ≤100	In-hospital	1.13 (1.01, 1.26)	Age, sex, BMI, SBP, DBP, smoking, drinking status, hypertension, diabetes mellitus, previous ICH, medication history, creatinine, GCS score
Mustanoja et al. (18) (Finland, RCS)	964, 550/414	66 ± 13	7 (3,14)	14 (10,15)	7.3 (2.7, 16)	Per Quartile increase	In-hospital	0.55 (0.32, 0.95)	Age, NIHSS, GCS, ICH volume, IVH, Statin use
							3-month	0.81 (0.54, 1.21)	
Ramírez-Moreno et al. (24) (Spain, PCS)	88, 50/38	73.8 ± 8.9	10.2 ± 7.6	13.0 ± 3.1	24.9 ± 35.0	>100 vs. ≤100	3-month	0.33 (0.11, 0.96)	Age, sex, hypertension, prior antihypertensive treatment, prior anticoagulation, ICH volume, ventricular extension, GCS, NIHSS, glucose
Rodriguez-Luna et al. (19) (Spain, PCS)	108, 62/46	71.6 ± 11.5	17 (10,20)	15 (11, 15)	27.4 ± 33.2	≥95 vs. <95	3-month	0.16 (0.03, 0.78)	Age, baseline ICH volume, intraventricular extension
Wen et al. (25) (China, PCS)	4606, 3087/1519	61.7 (51.9, 72.8)	9 (3,18)	15 (11,15)	NR	>100 vs. ≤100	3-month	0.54 (0.38, 0.78)	Age, sex, lipid-lowering drugs
Yang et al. (26) (China, RCS)	786, 486/300	59 (51, 68)	8 (4,12)	NR	15-45	Per 1 unit increase	In-hospital	1.47 (1.07, 2.01)	Age, NIHSS, Bleeding volume, Blood glucose, Serum Albumin, Fasting, Bleeding position, SBP lowering
You et al. (27) (China, PCS)	356, 236/120	64.1 ± 13.7	6 (3,10)	NR	9.3 (4.9, 20.0)	Per 1 unit increase	3-month	0.27 (0.08, 0.97)	Age, Gender, Smoking, hypertension, diabetes mellitus, Stroke, SBP, DBP, TC, TG, HDL-C, NIHSS, Bleeding volume

F, female; M, male; NR, not reported; PCS, prospective clinical study; RCS, retrospective clinical study; ICH, intracerebral hemorrhage; NIHSS, National Institutes of Health Stroke Scale; DBP, diastolic blood pressure; SBP, systolic blood pressure; CAD, Coronary artery disease; CHF, Congestive heart failure; CKD, Chronic kidney disease; BMI, body mass index; GCS, Glasgow coma scale; IVH, intraventricular hemorrhage; LDL-C, low-density lipoprotein cholesterol; TC, total cholesterol; TG, triglyceride; HDL-C, high-density lipoprotein cholesterol.

Most studies were rated with NOS scores ranging between 7 and 9 stars (Supplementary Table S4), with six studies scoring ≥8 stars, indicating all finally included studies were of high quality. The main sources of bias in these studies were recall bias and confounding bias.

### 3.3. Results of the in-hospital and 3-month mortality risk assessments

**Figure 2:**
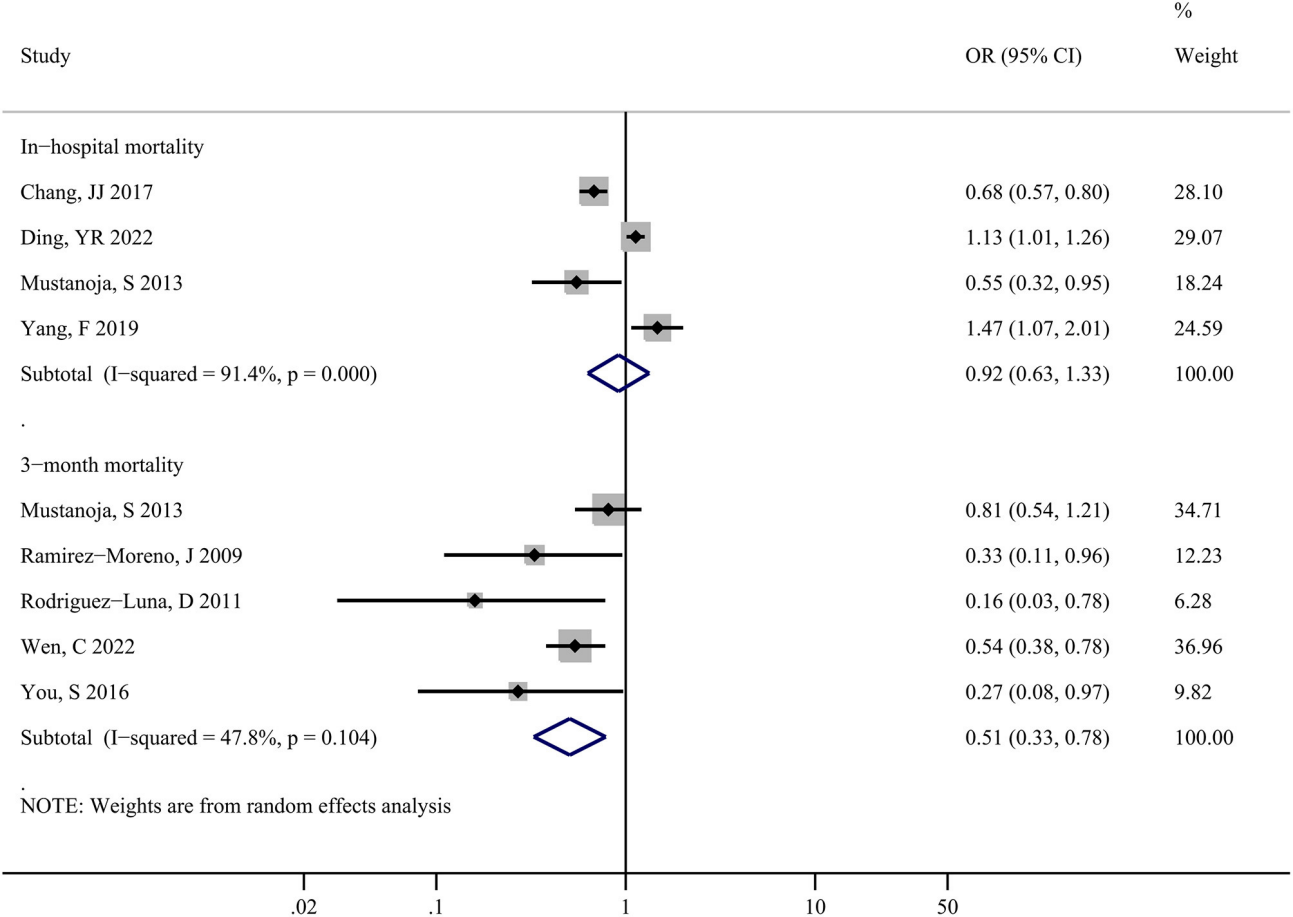
Forest plots showing pooled odds ratios (ORs) with 95% confidence intervals of in-hospital and 3-month mortality risk comparing the higher vs. lower serum LDL-C level in patients with sICH.

Figure 2 shows the forest plot for in-hospital and 3-month mortality risks. Four studies including 77, 855 patients, estimated the risk of in-hospital mortality and the patients' LDL-C levels, indicating no significant association between the elevated LDL-C levels and risks of in-hospital mortality (OR: 0.92; 95%CI: 0.63-1.33; *P* = 0.647) in sICH patients, with substantial heterogeneity across studies (I^2^ = 91.4%; *P* < 0.001).

Moreover, five studies including 6,122 patients showed that alteration in LDL-C levels and the risk of 3-month mortality had a strong association, with a pooled risk estimate of 0.51 (0.33, 0.78; *P* = 0.002) and no statistically significant heterogeneity (I^2^ = 47.8%; *P* = 0.104) across studies. In summary, a higher serum LDL-C level may have a beneficial effect on decreasing the risk of death in sICH patients.

### 3.4. Results of subgroup analyses

For the in-hospital mortality, the pooled results of the three subgroups (China, PCS, and RCS) were not statistically significant and highly heterogeneous (*P* > 0.05; I^2^ > 50%). However, the pooled results for the Western subgroup were significant (OR: 0.67; 95%CI: 0.57–0.78; *P* < 0.001), with almost no heterogeneity among the studies (I^2^ = 0%, *P* = 0.466) (Table 2).

**Table 2:** Subgroup analyses of baseline LDL-C level with the risk of in-hospital mortality and 3-month mortality among sICH patients.

**Outcomes**	**No. of study**	**OR (95%CI)**	***P*-value**	**Heterogeneity test**	
				**I**^2^ **(%)**	**P**_H_
**In-hospital mortality**					
Overall	4	0.92 (0.63, 1.33)	0.647	91.4	<0.001
Area					
China	2	1.23 (0.97, 1.57)	0.090	58.0	0.123
Western	2	0.67 (0.57, 0.78)	<0.001	0	0.466
Design					
PCS	2	0.88 (0.54, 1.45)	0.616	95.9	<0.001
RCS	2	0.92 (0.35, 2.42)	0.870	89.4	0.002
**3-month mortality**					
Overall	5	0.51 (0.33, 0.78)	0.002	47.8	0.104
Area					
China	2	0.50 (0.32, 0.77)	0.002	8.7	0.295
Western	3	0.44 (0.17, 1.12)	0.085	63.4	0.065
Design					
PCS	4	0.43 (0.28, 0.66)	<0.001	12.3	0.331
RCS	1	0.81 (0.54, 1.21)	0.306	NA	NA

OR, odds ratio; CI, confidence interval; PCS, prospective clinical study; RCS, retrospective clinical study; NA, not available.

For the 3-month mortality, the pooled estimates and size of heterogeneity for both subgroups of China and PCS were consistent with the original pooled results, whereas the pooled results for the Western and RCS subgroups were not significant (*P* > 0.05) (Table 2).

Overall, the study design variation did not have any effects on the LDL-C level's association with the in-hospital mortality risks of sICH patients, unlike the association with the 3-month mortality risk. Interestingly, the region of the study conducted seemed to influence the association of LDL-C level modulation with either in-hospital or 3-month mortality risk in sICH individuals. In other words, a higher LDL-C level may be associated with decreased in-hospital mortality risks in the West and 3-month mortality risks in China.

### 3.5. Sensitivity analysis and publication bias

As shown in Table 3, the sensitivity analysis revealed that the pooled risks were significant for both in-hospital and 3-month mortality risks in sICH, and no major change was observed in the pooled estimation even if any individual study was eliminated at each turn. Moreover, the change in the pooled effect size for in-hospital mortality risks, from 0.78 (0.51, 1.22) to 1.05 (0.82, 1.52), was not significant (*P* > 0.05). Likewise, the leave-out one study sensitivity analysis of 3-month mortality also indicated a significant (*P* < 0.05) stable pooled OR (95%CI), ranging from 0.47 (0.34, 0.65) to 0.62 (0.44, 0.89).

**Table 3:** Outcomes of the sensitivity analysis and test of publication bias.

**Outcomes**	**No. of studies**	**Sensitivity analysis**		**Egger's test**
		**OR (95% CI)**	**Robust**	***P*** **-value**
In-hospital mortality	4	0.78 (0.51, 1.22) to 1.05 (0.82, 1.52)	Yes	0.724
3-month mortality	5	0.47 (0.34, 0.65) to 0.62 (0.44, 0.89)	Yes	0.100

OR, odds ratio; CI, confidence interval.

The Egger's test could not identify any publication bias for both in-hospital (*P* = 0.724) and 3-month mortality (*P* = 0.100) risks for analyzed studies (Table 3). The funnel plot asymmetry analysis could not be performed for each subgroup due to the inclusion of fewer than 10 articles (28).

## 4. Discussion

This comprehensive meta-analysis of eight cohort studies involved 83,013 individuals with ICH to correlate the mortality risks of these patients with serum LDL-C levels. The sICH patients exhibited a robust influence of baseline LDL-C levels on the 3-month mortality risks, whereas no discernible effect was noticed for in-hospital mortality risk prediction. Sensitivity analyses also repeatedly confirmed this finding. However, values of LDL-C levels in predicting 3-month mortality risks were not significant in both the western and retrospective study subgroups, yet became statistically significant in the western subgroup when predicting in-hospital mortality risks, which might be respectively explained by significantly higher heterogeneity and a limited number of eligible studies. Thus, larger sample sizes are required to validate the extrapolation of these findings.

Furthermore, considerably higher heterogeneity (I^2^ = 91.4%) might mask the exact association of altered LDL-C levels with in-hospital mortality risks (OR: 0.92; 95%CI: 0.63–1.33). To overcome that, we conducted subgroup and sensitivity analyses, revealing no considerable heterogeneity without any adjustment for significance thresholds. The observed heterogeneity for in-hospital mortality risk might stem, in part, from a broad range of confounding factors that different studies had adjusted for at the cost of power reduction. Besides, the heterogeneity might correlate with participants' characteristics (e.g., age, geographic, and racial differences), as well as genetic polymorphisms (e.g., allelic variations of *APOE, PMF1*, and *SLC25A44* genes) (29–31). Also, differences in threshold values of LDL-C, diet, and exercise habits across studies could introduce heterogeneity. Interestingly, the Western subgroup analysis showed that the in-hospital mortality risk had a significant association with patients' LDL-C levels (OR: 0.67; 95%CI: 0.57–0.78). Pooled results of subgroups, however, showed inconsistency with the original results, suggesting that more high-quality studies are needed to establish this association, irrespective of the study area and participants' characteristics.

The effect of serum LDL-C level on ICH mortality risk may vary depending on the follow-up duration, which could partially explain the observed association of LDL-C level variation with the 3-month mortality risk but not in-hospital mortality, which could be related to severe ICH-associated higher mortality than LDL-C-induced ICH risk in the early stage. Hence, under aggravated ICH conditions, the correlation between mortality risks and LDL-C levels may not be crucially important (17, 32). It's noted that for patients with GCS scores between 9 and 15, low LDL-C levels can exacerbate hematoma expansion and in-hospital mortality risk, but not in coma patients (GCS scores 3–8). Most studies had a maximum follow-up period of 3 months, so the predictive value of LDL-C level for 1-year mortality could not be estimated in this meta-analysis.

Above all, our findings indicated that sICH patients having lower serum LDL-C levels could be at higher mortality risks. Our study supported the potential of LDL-C level alteration as an independent predictor for 3-month mortality risks in sICH patients and suggested outlines for risk stratification and clinical outcomes in ICH in designing patient-specific therapy.

### 4.1. Possible explanations for underlying mechanisms

Several different explanations regarding underlying mechanisms may be considered in this context.

First, LDL-C plays key functions in many physiological processes, such as maintaining vessel wall integrity. Reportedly, most healthy individuals are born with LDL-C levels ranging from 40 to 60 mg/dL (33). While lower LDL-C levels can be independently related to brain microhemorrhages under acute ICH conditions (34), which can further contribute to poor treatment response, stroke recurrence (35), and perihematomal edema expansion (36). The possible mechanisms are as follows: (1) disintegration of the endothelium (37); (2) necrotic death of medial smooth muscle cells (MSMCs) (38); (3) inhibited platelet aggregation (39); (4) accelerated osmotic membrane rupture in erythrocytes (40), and (5) impaired synaptic reconstruction (41). ICH patients with genetically reduced levels of LDL-C are more likely to carry APOE ε2/ε4 allele, indicating recurrent ICH (42, 43). Theoretically, high LDL-C levels could rescue ICH patients from hematoma enlargement and mortality risks. Further prospective studies are essential to assess an optimum LDL-C level to prevent adverse circumstances in ICH.

Second, the inclusion of observational studies prevented establishing a causal relationship in this meta-analysis. An excessively low LDL-C level can reversibly increase the mortality risk (44), but it may not necessitate a causal relationship in all cases (25). Whether LDL-C levels change over time due to physiological and clinical reasons is still unclear. A longitudinal study shows a subacute reduction in LDL-C levels preceding sICH (45), and both LDL-C and triglyceride levels can decline at the disease onset and restore to normal levels during the recovery phase (46–48). Debilitation and/or disease also can lower LDL-C levels (49, 50), which might be a surrogate for malnutritional or a sign of severe disease (51, 52), thus predisposing the individual to increased stroke mortality (53), indicating lower LDL-C level could induce chronic health problems and higher mortality, especially in the elderly (54). Hypocholesterolemia can lead to sepsis, adrenal failure, and increased mortality risks in critically ill patients (46). It is hypothesized that the pathophysiological abnormality of ICH patients causes higher mortality and reduction in LDL-C levels, in parallel, which may not be rescued by treatments directed to normalizing a single dysfunction (55). Thus, adjusting the LDL-C level to an optimum may not likely modify the death risk in sICH subjects. Furthermore, elderly pre-ICH statin users are at higher risk of comorbidities due to their lower LDL-C levels, just like individuals under antithrombic medications with an elevated risk of bleeding and stroke (56). Due to insufficient data on pre-ICH statin use, we could not evaluate the relationships between statins, sICH outcomes, and low LDL-C levels. However, we investigated the relationship between sICH outcomes and serum LDL-C levels. Besides, the pre-statin exposure rate among sICH is relatively low, without any independent correlation with worse treatment responses in sICH patients (18, 57), which is consistent with other meta-analyses suggesting that pre-ICH statin application might not have any association with post-ICH mortality risks (56, 58–60). Above all, the reciprocal correlation between the LDL-C level at admission and mortality risks in sICH patients should be interpreted with caution. Further large-scale studies are needed to further elucidate the impact of low LDL-C levels and ICH death risk.

### 4.2. Statin treatment after ICH

Although conventionally lowering lipid levels is considered the best to control atherosclerotic cardiovascular diseases (61), the effect of statins on optimally regulating LDL-C levels still needs more validation to gauge the risk of sICH in atherosclerotic patients. Since chronic use of statins increases bleeding complications, while its sudden discontinuation can predispose to ischemia, the application of statins in acute sICH patients is highly debated (62). Similarly, a recent study (10) advised against abruptly discontinuing statin medication in cases of acute ICH without first conducting a thorough health assessment. In contrast, a prior Markov decision analysis endorsed that avoiding statins is expedient in all ICH survivors after weighing these competing risks and benefits (63). However, individualized treatment decisions based on expert consultation have been advocated by recent guidelines (64). Furthermore, due to the limited data availability, our meta-analysis was unable to identify the threshold value, and we could not comprehend the linear or non-linear association of serum LDL-C with mortality risk in sICH patients. The previous report showed low LDL-C levels (<100 mg/dL) containing subjects had enhanced 3-month mortality and were more prone to hematoma enlargement than their counterparts with higher LDL-C levels (110–129 mg/dL) (25). Particularly, <70 mg/dL can increase the risk of hematoma expansion and in-hospital mortality by several folds compared to subjects with higher LDL-C levels (17, 19, 65). Statin treatment in ICH patients with relatively low LDL-C should be approached with caution due to the increased lifetime risk. Hence, randomized controlled trials are urgently necessary to resolve the complicacy of statin use in patients with higher risks of ICH.

### 4.3. Strengths and limitations

We found that the elevated level of LDL-C exhibits an inverse relationship with the in-hospital and 3-month mortality risks, respectively, among post-ICH patients in western countries and China. To the best of our knowledge, this is the first meta-analysis reporting an assessment of LDL-C level variation in relation to sICH mortality risk. The strengths of the study include its extensive literature search, stringent criteria, and thorough scrutinization of included studies. Despite the limited number of articles, the sample size was considerably large, and the accuracy of the meta-analysis results was relatively satisfactory. Moreover, the methodological quality control for included reports matched the international standard. Despite the influence of confounding factors, like pre-treatment medications, ICH volumes, admission NIHSS scores, and hypertension, all studies produced highly objective and reasonable observations after multiple factor adjustments. Furthermore, the sensitivity analyses and Egger's test suggested the good reproducibility and high credibility of the pooled results across the included studies. Finally, we strictly adhered to the PRISMA recommendations for study screening, data extraction, quality control checking, and analyses to reduce recall bias, especially for observational studies.

Yet, we note that this review does have some limitations. First, being a study-level meta-analysis, this study could not consider methodological variations in analyzed articles. It wasn't possible to adopt a uniform adjustment of variables for all studies. Also, a standardized analysis of the age and sex of the participants could not be performed to identify potential confounding factors for heterogeneity. An individual-level meta-analysis will be appropriate to evaluate the relationship between ICH mortality risks and low LDL-C levels in terms of demographics, existing medications, and comorbidities.

Second, in all cases, only one-time measured post-ICH serum LDL-C level values were included for analysis, which might introduce classification bias. There were no pre-ICH onset data for these patients, and possible variations in LDL-C levels during the hospital stay and follow-up periods were not considered. It remains unclear whether an acute sICH condition induces LDL-C level alteration and/or whether the reverse phenomenon is the actual culprit in elevating the mortality risk. Time-average serum LDL-C level analysis can be useful to verify the association of pathological LDL-C levels with higher mortality risks.

Third, different cutoffs for LDL-C levels were used in the prediction of mortality risks across studies, and the methodological heterogeneity might lead to the discrepancy in results, which prevented us from determining the optimal value of LDL-C level in better predicting ICH survival. And the enrolled studies were ineligible for the dose-response analysis because they lacked the necessary data (e.g., the number of cases in each LDL-C level). Additionally, abnormally low LDL-C levels might have underpowered our results. We expect future studies to analyze clinically uncommon subsets of patients critically for better understanding.

Fourth, all eligible studies involved cohort analyses, which had a higher susceptibility to bias from confounding factors than randomized controlled trials. Residual confounding biases are sometimes unavoidable due to the omission of or insufficiently measured variables. Some important confounding factors related to mortality risk should be adjusted as comprehensively and consistently as possible in the statistical model, while the severity of ICH (GCS and NIHSS scores), hypertension, pre-medications, divergent inciting causes of ICH (blood pressure, cerebral amyloid angiopathy, anticoagulation, and vascular anomalies), complete neuroimaging data (hematoma shape, volume, and site), and time from ICH occurrence to cranial CT scan were not fully captured in several of these studies. Inadequate adjustment may have resulted in an overestimation of the risk estimate.

Fifth, most participants were Han Chinese and Caucasians, which limited the generalizability of our observations to the general population, but it might have some reference values. Caution should be paid to extrapolating these results to other ethnic groups. The association and optimal range of LDL-C level change may differ across ethnic groups due to their varying baseline LDL-C levels, environments, and individual risk factors.

## 5. Conclusions

In conclusion, an increase in LDL-C level is inversely associated with 3-month mortality risks in sICH patients but not significantly correlated with in-hospital mortality risks. Serum LDL-C level can be a potential independent biomarker of mortality risk evaluation in sICH patients and may be helpful in early decision-making in clinical practices and contribute to identifying those at higher risks of mortality. Nevertheless, the subgroup analyses revealed inconsistencies with the original pooled results, indicating further well-designed studies with stringent quality control and larger sample sizes are recommended to validate the stability and extrapolation of these results as well as to determine an appropriate LDL-C range.

## Data availability statement

The original contributions presented in the study are included in the article/Supplementary material, further inquiries can be directed to the corresponding author.

## Author contributions

XS and JL were involved in the conception and design of the study. JL, GL, and YZ contributed to the literature screening, data acquisition, statistical analysis, and interpretation. JL and GC wrote the manuscript, which was revised, and approved by all the authors for publication. XS and JZ participated in the review, editing, and supervision of the article. All authors contributed to the study and approved the submitted manuscript.

## References

[1] Tsao CW Aday AW Almarzooq ZI Alonso A Beaton AZ Bittencourt MS . Heart disease and stroke statistics-2022 update: a report from the american heart association. Circulation. (2022) 145:e153–639. 10.1161/CIR.0000000000001052 35078371

[2] An SJ Kim TJ Yoon B-W. Epidemiology, risk factors, and clinical features of intracerebral hemorrhage: an update. J Stroke. (2017) 19:3–10. 10.5853/jos.2016.00864 28178408 PMC5307940

[3] Krishnamurthi RV Feigin VL Forouzanfar MH Mensah GA Connor M Bennett DA . Global and regional burden of first-ever ischaemic and haemorrhagic stroke during 1990–2010: findings from the global burden of disease study 2010. Lancet Global Health. (2013) 1:e259–e81. 10.1016/S2214-109X(13)70089-5 25104492 PMC4181351

[4] Cordonnier C Demchuk A Ziai W Anderson CS. Intracerebral haemorrhage: current approaches to acute management. Lancet. (2018) 392:1257–68. 10.1016/S0140-6736(18)31878-6 30319113

[5] Hemphill JC Greenberg SM Anderson CS Becker K Bendok BR Cushman M . Guidelines for the management of spontaneous intracerebral hemorrhage. Stroke. (2015) 46:2032–60. 10.1161/STR.0000000000000069 26022637

[6] Gittler M Davis AM. Guidelines for adult stroke rehabilitation and recovery. JAMA. (2018) 319:820–1. 10.1001/jama.2017.22036 29486016

[7] Valdes-Marquez E Parish S Clarke R Stari T Worrall BB Hopewell JC. Relative Effects of Ldl-C on Ischemic Stroke and Coronary Disease. Neurology. (2019) 92:e1176. 10.1212/WNL.0000000000007091 30787162 PMC6511103

[8] Ference BA Ginsberg HN Graham I Ray KK Packard CJ Bruckert E . Low-density lipoproteins cause atherosclerotic cardiovascular disease. Eur Heart J. (2017) 38:2459–72. 10.1093/eurheartj/ehx144 28444290 PMC5837225

[9] Wadhera RK Steen DL Khan I Giugliano RP Foody JM A. Review of low-density lipoprotein cholesterol, treatment strategies, and its impact on cardiovascular disease morbidity and mortality. J Clin Lipidol. (2016) 10:472–89. 10.1016/j.jacl.2015.11.010 27206934

[10] Shoamanesh A Selim M. Use of lipid-lowering drugs after intracerebral hemorrhage. Stroke. (2022) 53:2161–70. 10.1161/STROKEAHA.122.036889 35658483 PMC9248990

[11] Falcone GJ Kirsch E Acosta JN Noche RB Leasure A Marini S . Genetically elevated ldl associates with lower risk of intracerebral hemorrhage. Ann Neurol. (2020) 88:56–66. 10.1002/ana.25740 32277781 PMC7523882

[12] Sun L Clarke R Bennett D Guo Y Walters RG Hill M . Causal associations of blood lipids with risk of ischemic stroke and intracerebral hemorrhage in chinese adults. Nat Med. (2019) 25:569–74. 10.1038/s41591-019-0366-x 30858617 PMC6795549

[13] Wang X Dong Y Qi X Huang C Hou L. Cholesterol levels and risk of hemorrhagic stroke: a systematic review and meta-analysis. Stroke. (2013) 44:1833–9. 10.1161/strokeaha.113.001326 23704101

[14] Ma C Na M Neumann S Gao X. Low-density lipoprotein cholesterol and risk of hemorrhagic stroke: a systematic review and dose-response meta-analysis of prospective studies. Curr Atheroscler Rep. (2019) 21:52. 10.1007/s11883-019-0815-5 31748963

[15] Jin X Chen H Shi H Fu K Li J Tian L . Lipid levels and the risk of hemorrhagic stroke: a dose–response meta-analysis. Nutr Metab Cardiovas Dis. (2021) 31:23–35. 10.1016/j.numecd.2020.10.014 33257190

[16] Chang JJ Katsanos AH Khorchid Y Dillard K Kerro A Burgess LG . Higher low-density lipoprotein cholesterol levels are associated with decreased mortality in patients with intracerebral hemorrhage. Atherosclerosis. (2018) 269:14–20. 10.1016/j.atherosclerosis.2017.12.008 29253643

[17] Ding Y Wang Y Liu L Gu H Yang K Li Z . Combined association of low-density lipoprotein cholesterol levels and systolic blood pressure to the outcome of intracerebral hemorrhage: data from the china stroke center alliance. Oxid Med Cell Longev. (2022) 2022:6206315. 10.1155/2022/6206315 35761874 PMC9233602

[18] Mustanoja S Strbian D Putaala J Meretoja A Curtze S Haapaniemi E . Association of prestroke statin use and lipid levels with outcome of intracerebral hemorrhage. Stroke. (2013) 44:2330–2. 10.1161/STROKEAHA.113.001829 23760210

[19] Rodriguez-Luna D Rubiera M Ribo M Coscojuela P Pagola J Piñeiro S . serum low-density lipoprotein cholesterol level predicts hematoma growth and clinical outcome after acute intracerebral hemorrhage. Stroke. (2011) 42:2447–52. 10.1161/STROKEAHA.110.609461 21799167

[20] Page MJ McKenzie JE Bossuyt PM Boutron I Hoffmann TC Mulrow CD . The prisma 2020 statement: an updated guideline for reporting systematic reviews. BMJ. (2021) 372:n71. 10.1136/bmj.n71 33782057 PMC8005924

[21] Wells G Shea B O'Connell J. The Newcastle-Ottawa Scale (Nos) for Assessing the Quality of Nonrandomised Studies in Meta-Analyses. Ottawa Health Research Institute Web site (2014).

[22] Higgins JPT. Measuring inconsistency in meta-analyses. BMJ. (2003) 327:557–60. 10.1136/bmj.327.7414.557 12958120 PMC192859

[23] Egger M Smith GD Schneider M Minder C. Bias in meta-analysis detected by a simple, graphical test. BMJ. (1997) 315:629–34. 10.1136/bmj.315.7109.629 9310563 PMC2127453

[24] Ramírez-Moreno JM Casado-Naranjo I Portilla JC Calle ML Tena D Falcón A . Serum cholesterol ldl and 90-day mortality in patients with intracerebral hemorrhage. Stroke. (2009) 40:1917–20. 10.1161/STROKEAHA.108.536698 19299638

[25] Wen C-P Lee Y-C Sun Y-T Huang C-Y Tsai C-H Chen P-L . Low-Density Lipoprotein Cholesterol and Mortality in Patients with Intracerebral Hemorrhage in Taiwan. Front Neurol. (2022) 12:2377. 10.3389/fneur.2021.793471 35113980 PMC8802633

[26] Yang F Sun M Wang L Li S Guo X Dou J . The association between blood pressure decreasing rates and survival time in patients with acute intracerebral hemorrhage. J Neurol Sci. (2019) 406:116449. 10.1016/j.jns.2019.116449 31654959

[27] You S Zhong C Xu J Han Q Zhang X Liu H . Ldl-C/Hdl-C ratio and risk of all-cause mortality in patients with intracerebral hemorrhage. Neurol Res. (2016) 38:903–8. 10.1080/01616412.2016.1204797 27412564

[28] Sedgwick P Marston L. How to read a funnel plot in a meta-analysis. BMJ. (2015) 351:h4718. 10.1136/bmj.h4718 26377337

[29] Devan WJ Falcone GJ Anderson CD Jagiella JM Schmidt H Hansen BM . Heritability estimates identify a substantial genetic contribution to risk and outcome of intracerebral hemorrhage. Stroke. (2013) 44:1578–83. 10.1161/STROKEAHA.111.000089 23559261 PMC3684199

[30] Carpenter AM Singh IP Gandhi CD Prestigiacomo CJ. Genetic risk factors for spontaneous intracerebral haemorrhage. Nat Rev Neurol. (2016) 12:40–9. 10.1038/nrneurol.2015.226 26670299

[31] Woo D. Falcone Guido J, Devan William J, Brown WM, Biffi A, Howard Timothy D, et al. Meta-analysis of genome-wide association studies identifies 1q22 as a susceptibility locus for intracerebral hemorrhage. Am J Hum Genetics. (2014) 94:511–21. 10.1016/j.ajhg.2014.02.012 24656865 PMC3980413

[32] Wang Y Wu J Gu H Yang K Jiang R Li Z . Lower low-density lipoprotein cholesterol levels are associated with an increased risk of hematoma expansion and ensuing mortality in acute ich patients. Neurol Sci. (2022) 43:3121–9. 10.1007/s10072-021-05742-w 34806117

[33] Ference BA Graham I Tokgozoglu L Catapano AL. Impact of lipids on cardiovascular health: JACC health promotion series. J Am Coll Cardiol. (2018) 72:1141–56. 10.1016/j.jacc.2018.06.046 30165986

[34] Lee S-H Bae H-J Yoon B-W Kim H Kim D-E Roh J-K. Low concentration of serum total cholesterol is associated with multifocal signal loss lesions on gradient-echo magnetic resonance imaging: analysis of risk factors for multifocal signal loss lesions. Stroke. (2002) 33:2845–9. 10.1161/01.STR.0000036092.23649.2E 12468780

[35] Wang D-N Hou X-W Yang B-W Lin Y Shi J-P Wang N. Quantity of cerebral microbleeds, antiplatelet therapy, and intracerebral hemorrhage outcomes: a systematic review and meta-analysis. J Stroke Cerebrovasc Dis. (2015) 24:2728–37. 10.1016/j.jstrokecerebrovasdis.2015.08.003 26342996

[36] Lin W-M Yang T-Y Weng H-H Chen C-F Lee M-H Yang J-T . Brain microbleeds: distribution and influence on hematoma and perihematomal edema in patients with primary intracerebral hemorrhage. Neuroradiol J. (2013) 26:184–90. 10.1177/197140091302600208 23859241 PMC5228727

[37] Bang OY Saver JL Liebeskind DS Starkman S Villablanca P Salamon N . Cholesterol level and symptomatic hemorrhagic transformation after ischemic stroke thrombolysis. Neurology. (2007) 68:737–42. 10.1212/01.wnl.0000252799.64165.d5 17182976

[38] Ooneda G Yoshida Y Suzuki K Shinkai H Hori S Kobori K . Smooth muscle cells in the development of plasmatic arterionecrosis, arteriosclerosis, and arterial contraction. Blood Vessels. (1978) 15:148–56. 10.1159/000158160 630129

[39] Chui DH Marotta F Rao ML Liu DS Zhang SC Ideo C. Cholesterol-rich Ldl perfused at physiological ldl-cholesterol concentration induces platelet aggregation and paf-acetylhydrolase activation. Biomed Pharmacother. (1991) 45:37–42. 10.1016/0753-3322(91)90152-J 2043757

[40] Yamori Y Nara Y Horie R Ooshima A. Abnormal membrane characteristics of erythrocytes in rat models and men with predisposition to stroke. Clin Exp Hypertens. (1980) 2:1009–21. 10.3109/10641968009037158 6256139

[41] Goritz C Mauch DH Pfrieger FW. Multiple mechanisms mediate cholesterol-induced synaptogenesis in a cns neuron. Mol Cell Neurosci. (2005) 29:190–201. 10.1016/j.mcn.2005.02.006 15911344

[42] Raffeld MR Biffi A Battey TWK Ayres A Viswanathan A Greenberg S . Apoe E4 and lipid levels affect risk of recurrent nonlobar intracerebral. Hemorrhage. 85, 349–56. (2015). 10.1212/WNL.0000000000001790 26115730 PMC4520812

[43] Sawyer RP Sekar P Osborne J Kittner SJ Moomaw CJ Flaherty ML . Racial/ethnic variation of alleles for lobar intracerebral hemorrhage. Neurology. (2018) 91:e410–e20. 10.1212/WNL.0000000000005908 29959260 PMC6093767

[44] Sung K-C Huh JH Ryu S Lee J-Y Scorletti E Byrne CD . Low levels of low-density lipoprotein cholesterol and mortality outcomes in non-statin users. J Clin Med. (2019) 8:10. 10.3390/jcm8101571 31581520 PMC6832139

[45] Phuah C-L Raffeld MR Ayres AM Viswanathan A Greenberg SM Biffi A . Subacute decline in serum lipids precedes the occurrence of primary intracerebral hemorrhage. Neurology. (2016) 86:2034–41. 10.1212/WNL.0000000000002716 27164693 PMC4891207

[46] Liu Q Zhao W Xing Y Hong Y Zhou G. Low triglyceride levels are associated with unfavorable outcomes in patients with spontaneous intracerebral hemorrhage. Neurocrit Care. (2021) 34:218–26. 10.1007/s12028-020-01023-0 32557109

[47] Butterworth RJ Marshall WJ Bath PMW. Changes in serum lipid measurements following acute ischaemic stroke. Cerebrovascular Dis. (1997) 7:10–3. 10.1159/000108156

[48] Roquer J Campello AR Gomis M Ois A Munteis E Bohm P. Serum lipid levels and in-hospital mortality in patients with intracerebral hemorrhage. Neurology. (2005) 65:1198–202. 10.1212/01.wnl.0000180968.26242.4a 16247046

[49] Jacobs D Blackburn H Higgins M Reed D Iso H McMillan G . Report of the conference on low blood cholesterol: mortality associations. Circulation. (1992) 86:1046–60. 10.1161/01.CIR.86.3.1046 1355411

[50] Ranieri Renzo Rozzini Simone Franzo P. Serum cholesterol levels as a measure of frailty in elderly patients. Exp Aging Res. (1998) 24:169–79. 10.1080/036107398244300 9555569

[51] Iribarren C Jacobs D Sadler M Claxton A Sidney S. Low total serum cholesterol and intracerebral hemorrhagic stroke: is the association confined to elderly men? Kaiser Perm Med Care Program. (1996) 27:1993–8. 10.1161/01.STR.27.11.1993 8898804

[52] Davis JP Wong AA Schluter PJ Henderson RD O'Sullivan JD Read SJ. Impact of premorbid undernutrition on outcome in stroke patients. Stroke. (2004) 35:1930–4. 10.1161/01.STR.0000135227.10451.c9 15218159

[53] Gariballa SE Parker SG Taub N Castleden CM. Influence of nutritional status on clinical outcome after acute stroke. Am J Clin Nutr. (1998) 68:275–81. 10.1093/ajcn/68.2.275 9701183

[54] Lewington S Whitlock G Clarke R Sherliker P Emberson J Halsey J . Blood cholesterol and vascular mortality by age, sex, and blood pressure: a meta-analysis of individual data from 61 prospective studies with 55,000 vascular deaths. Lancet. (2007) 370:1829–39. 10.1016/S0140-6736(07)61778-4 18061058

[55] Johannesen CDL Langsted A Mortensen MB Nordestgaard BG. Association between low density lipoprotein and all cause and cause specific mortality in denmark: prospective cohort study. BMJ. 371:4266. 10.1136/bmj.m4266 33293274 PMC7722479

[56] Lei C Wu B Liu M Chen Y. Association between statin use and intracerebral hemorrhage: a systematic review and meta-analysis. Eur J Neurol. (2014) 21:192–8. 10.1111/ene.12273 24118228

[57] Priglinger M Arima H Anderson C Krause M. No relationship of lipid-lowering agents to hematoma growth: pooled analysis of the intensive blood pressure reduction in acute cerebral hemorrhage trials studies. Stroke. (2015) 46:857–9. 10.1161/STROKEAHA.114.007664 25657175

[58] Lei C Chen T Chen C Ling Y. Pre-intracerebral hemorrhage and in-hospital statin use in intracerebral hemorrhage: a systematic review and meta-analysis. World Neurosurg. (2018) 111:47–54. 10.1016/j.wneu.2017.12.020 29248775

[59] Jung J-M Choi J-Y Kim HJ Seo W-K. Statin use in spontaneous intracerebral hemorrhage: a systematic review and meta-analysis. Int J Stroke. (2015) 10:10–7. 10.1111/ijs.12624 26306674

[60] Biffi A Devan WJ Anderson CD Ayres AM Schwab K Cortellini L . Statin use and outcome after intracerebral hemorrhage: case-control study and meta-analysis. Neurology. (2011) 76:1581–8. 10.1212/WNL.0b013e3182194be9 21451150 PMC3100126

[61] Cannon CP. Low-density lipoprotein cholesterol: lower is totally better. J Am Coll Cardiol. (2020) 75:2119–21. 10.1016/j.jacc.2020.03.033 32209335

[62] Endres M Nolte CH Scheitz JF. Statin treatment in patients with intracerebral hemorrhage. Stroke. (2018) 49:240–6. 10.1161/STROKEAHA.117.019322 29191849

[63] Westover MB Bianchi MT Eckman MH Greenberg SM. Statin use following intracerebral hemorrhage: a decision analysis. Arch Neurol. (2011) 68:573–9. 10.1001/archneurol.2010.356 21220650 PMC3158138

[64] Shoamanesh A Patrice Lindsay M Castellucci LA Cayley A Crowther M de Wit K . Canadian stroke best practice recommendations: management of spontaneous intracerebral hemorrhage, 7th edition update 2020. Int J Stroke. (2021) 16:321–41. 10.1177/1747493020968424 33174815

[65] Elkhatib THM Shehta N Bessar AA. Hematoma expansion predictors: laboratory and radiological risk factors in patients with acute intracerebral hemorrhage: a prospective observational study. J Stroke Cereb Dis. (2019) 28:2177–86. 10.1016/j.jstrokecerebrovasdis.2019.04.038 31133486

